# The genus *Galumna* in Nepalese oribatid mite fauna, with notes on systematic placement of some species (Acari, Oribatida, Galumnidae)

**DOI:** 10.3897/zookeys.438.8192

**Published:** 2014-09-01

**Authors:** Sergey G. Ermilov, Jochen Martens, Andrei V. Tolstikov

**Affiliations:** 1 Tyumen State University, Tyumen, Russia; 2 Johannes Gutenberg University, Mainz, Germany

**Keywords:** Oribatida, new species, supplementary description, new combination, Galumnidae, *Galumna*, *Notogalumna*, *Dimidiogalumna*, Nepal

## Abstract

The oribatid mite genus *Galumna* (Oribatida, Galumnidae) is recorded for the first time in Nepal. A new species, *Galumna tetraporosa* **sp. n.**, is described from soil of secondary mixed broadleaved forest. It is most similar morphologically to *G. tokyoensis* Aoki, 1966 and *G. valida* Aoki, 1994, however, it differs from both by the absence of interlamellar setae and the presence of two pairs of notogastral porose areas *Aa. Galumna granalata* Aoki, 1984 is redescribed on the basis of specimens from Nepal. *Galumna floridae* (Jacot, 1929) and *G. hexagona* Balogh, 1960 are transferred to the genus *Notogalumna*; *G. mauritii* Mahunka, 1978 is transferred to the genus *Dimidiogalumna*.

## Introduction

*Galumna* is a genus that was proposed by Heyden (1826) with *Notaspis alatus* Hermann, 1804 as type species. Currently, it comprises more than 170 species having a cosmopolitan distribution (for example, Subías 2004, online version 2014). The main generic characters are summarized by Ermilov et al. (2013). An identification key to many species of *Galumna* has been presented by Balogh and Balogh (2002).

In the course of taxonomic identification of Nepalese *Galumna*, we discovered two species: *Galumna granalata* Aoki, 1984 and a new one. This genus is recorded for the first time in Nepal. The primary goal of this paper is to describe and illustrate a new species. The secondary goal is to make a supplementary description of *Galumna granalata* based on the Nepalese material. This species was described Aoki (1984) and is distributed in Japan and Taiwan (Subías 2004, online version 2014). The original description of *Galumna granalata* is incomplete (lacking information about the length of morphological structures, leg setation and solenidia, gnathosoma; only figures of the pteromorph and the dorsal side of the body present).

Additionally, the systematic placement of three species of the genus *Galumna*, *Galumna floridae* (Jacot, 1929), *Galumna hexagona* Balogh, 1960 and *Galumna mauritii* Mahunka, 1978, are discussed.

## Material and methods

Three specimens (holotype: male; two paratypes: one male and one female) of *Galumna tetraporosa* sp. n. are from: Nepal, 2450–2720 m a.s.l., Terhathum District, ridge Tinjura Dara, broadleaved forest, soil, 17.IX.1983, collected by J. Martens and B. Daams.

Eight specimens of *Galumna granalata* are from: Nepal, 1650–1800 m a.s.l., Taplejung District, valley of the Kabeli River, below village Yamputhin, secondary mixed broadleaved forest with bamboo, soil, 03–04.IX.1983, collected by J. Martens and B. Daams.

Specimens were mounted in lactic acid on temporary cavity slides for measurement and illustration. The body length was measured in lateral view, from the tip of the rostrum to the posterior edge of the ventral fig. The notogastral width refers to the maximum width in dorsal aspect. Lengths of body setae were measured in lateral aspect. All body measurements are presented in micrometers. Formulae for leg setation are given in parentheses according to the sequence trochanter–femur–genu–tibia–tarsus (famulus included). Formulae for leg solenidia are given in square brackets according to the sequence genu–tibia–tarsus. General terminology used in this paper follows that of Grandjean (summarized by Norton and Behan-Pelletier 2009).

## Taxonomy

### Galumna tetraporosa sp. n.

Taxon classificationAnimaliaOribatidaGalumnidae

http://zoobank.org/58AC6AB0-8289-46C9-8BA0-2CADF9DE6029

Figs 1 – 5

#### Diagnosis.

Body size: 913–979 × 730–763. Rostrum pointed. Rostral and lamellar setae of medium size. Interlamellar setae represented by alveolus. Bothridial setae long, clavate, barbed. Lamellar and sublamellar lines divergent in medio-distal part. Anterior notogastral margin not developed. Notogaster with five pairs of small, rounded porose areas, *Aa* divided into two parts. Median pore absent. Aggenital and ano-adanal setae minute. Postanal porose area present, elongated.

#### Description.

*Measurements*. Body length: 962 (holotype), 913–979 (two paratypes); notogaster width: 730 (holotype), 730–763 (two paratypes).

*Integument*. Body color black to dark brown. Body surface smooth. Pteromorphs with poorly visible radiate wrinkles.

*Prodorsum*. Rostrum with strong tooth (12–20). Rostral setae (*ro*, 53–65) setiform, barbed. Lamellar setae (*le*, 65–77) setiform, little thicker and less barbed than rostral setae. Interlamellar setae absent, represented alveolus. Bothridial setae (*ss*, 114–123) with long stalk and shorter, barbed clavate head. Exobothridial setae absent. Porose areas *Ad* present, elongate oval (24–32 × 12–16), but visible only in dissected specimen. Lamellar lines (*L*) curving backwards; sublamellar lines (*S*) parallel in basal part and divergent in medio-distal part to lamellar lines.

*Notogaster*. Anterior notogastral margin not developed. Dorsophragmata (*D*) of medium size, elongated. Notogastral setae represented by 10 pairs of alveoli. Five pairs of small, rounded porose areas with distinct borders: *Aa* divided into two porose areas – smaller lateral (8–16) and larger medial (20–28); *A1* (12–16) and *A2* (16–28) located close to each other; *A3* (24–36) usually largest. Alveoli *la* inserted posteriorly to *Aa*. Lyrifissures *im* located anteriorly or antero-laterally to *A1*. Opisthonotal gland openings (*gla*) located antero-laterally to *A2*. Median pore absent.

*Gnathosoma*. Generally, morphology of subcapitulum, palps and chelicerae typical for most Galumnidae (for example, see Ermilov and Anichkin 2010, 2011; Ermilov et al. 2011). Subcapitulum little longer than wide (196 × 192). Subcapitular setae setiform, slightly barbed; *a* (32–36) longer, more barbed and thicker than *m* (24) and *h* (16–20). Two pairs of adoral setae (*or*_1_, *or*_2_, 20) setiform, barbed. Palps (143) with setation 0–2–1–3–9(+ω). Solenidion straight, thickened, blunt-ended, attached to eupathidium. Chelicerae (246) with two barbed setae; *cha* (94) longer than *chb* (53). Trägårdh’s organ distinct.

*Epimeral and lateral podosomal regions*. Apodemes (1, 2, sejugal, 3) well visible. Four pairs of epimeral setae (*1b*, *3b*, *4a*, *4b*) observed ventrally, all setiform, thin, smooth, similar in length (24–32). Discidia (*dis*) triangular, circumpedal carinae (*cp*) distinct.

*Anogenital region*. Six pairs of genital setae (*g*_1_–*g*_6_, 24–28) setiform, thin, smooth. Anterior edge of genital figs with three setae. One pair of aggenital (*ag*), two pairs of anal (*an*_1_, *an*_2_) and three pairs of adanal (*ad*_1_–*ad*_3_) setae minute, similar in length (4). Adanal setae *ad*_3_ inserted laterally to adanal lyrifissures *iad*. Postanal porose area (*Ap*) elongated (36–49 × 8–12).

*Legs*. Generally, morphology of leg segments, setae and solenidia typical for most Galumnidae (for example, see Ermilov and Anichkin 2010, 2011; Ermilov et al. 2011). Formulae of leg setation and solenidia: I (1–4–3–4–20) [1–2–2], II (1–4–3–4–15) [1–1–2], III (1–2–1–3–15) [1–1–0], IV (1–2–2–3–12) [0–1–0]; homology of setae and solenidia indicated in Table 1.

**Figure 1.**
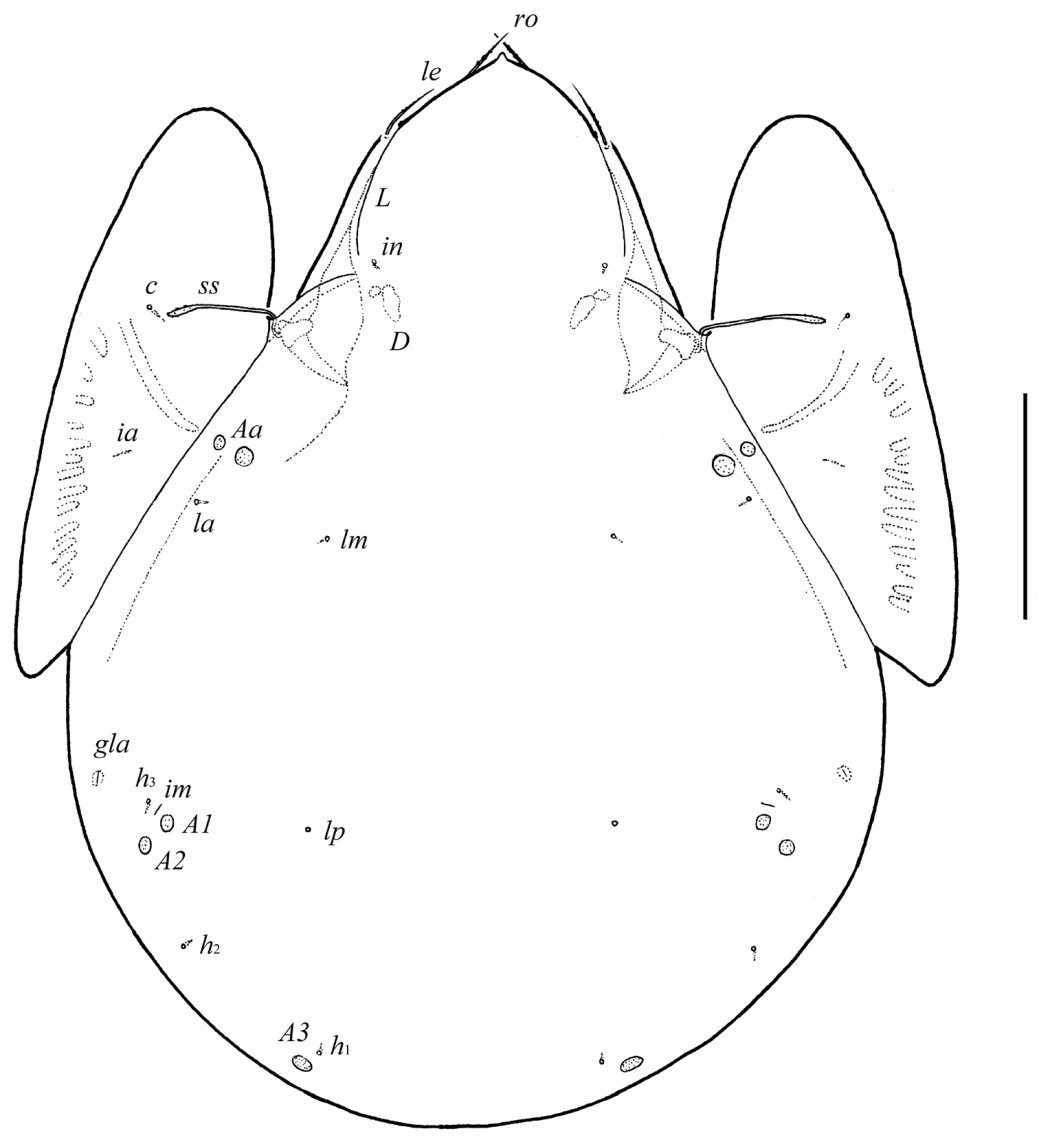
*Galumna tetraporosa* sp. n., adult: dorsal view. Scale bar 200 μm.

**Figure 2.**
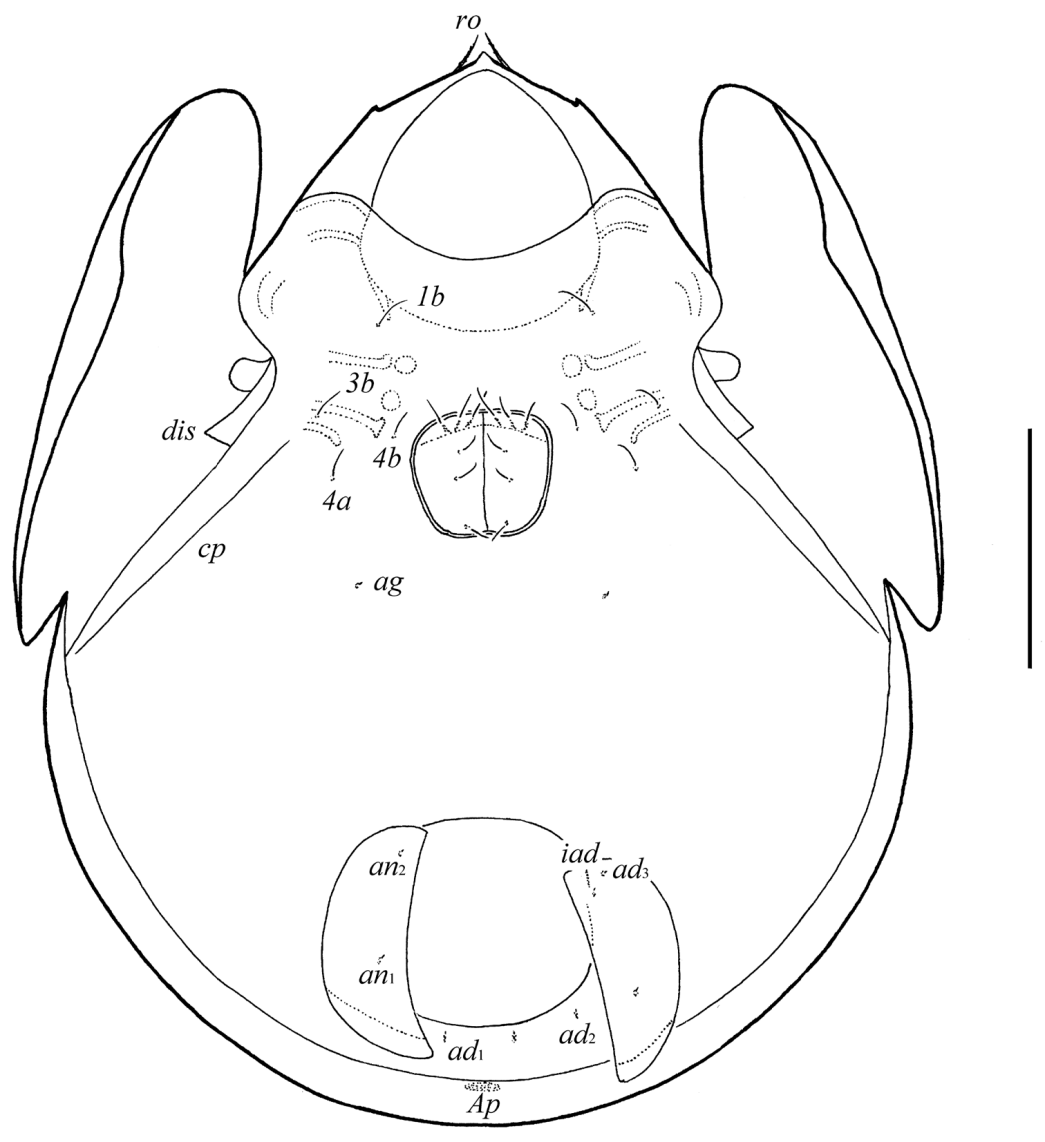
*Galumna tetraporosa* sp. n., adult: ventral view (gnathosoma and legs not illustrated). Scale bar 200 μm.

**Figures 3–5.**
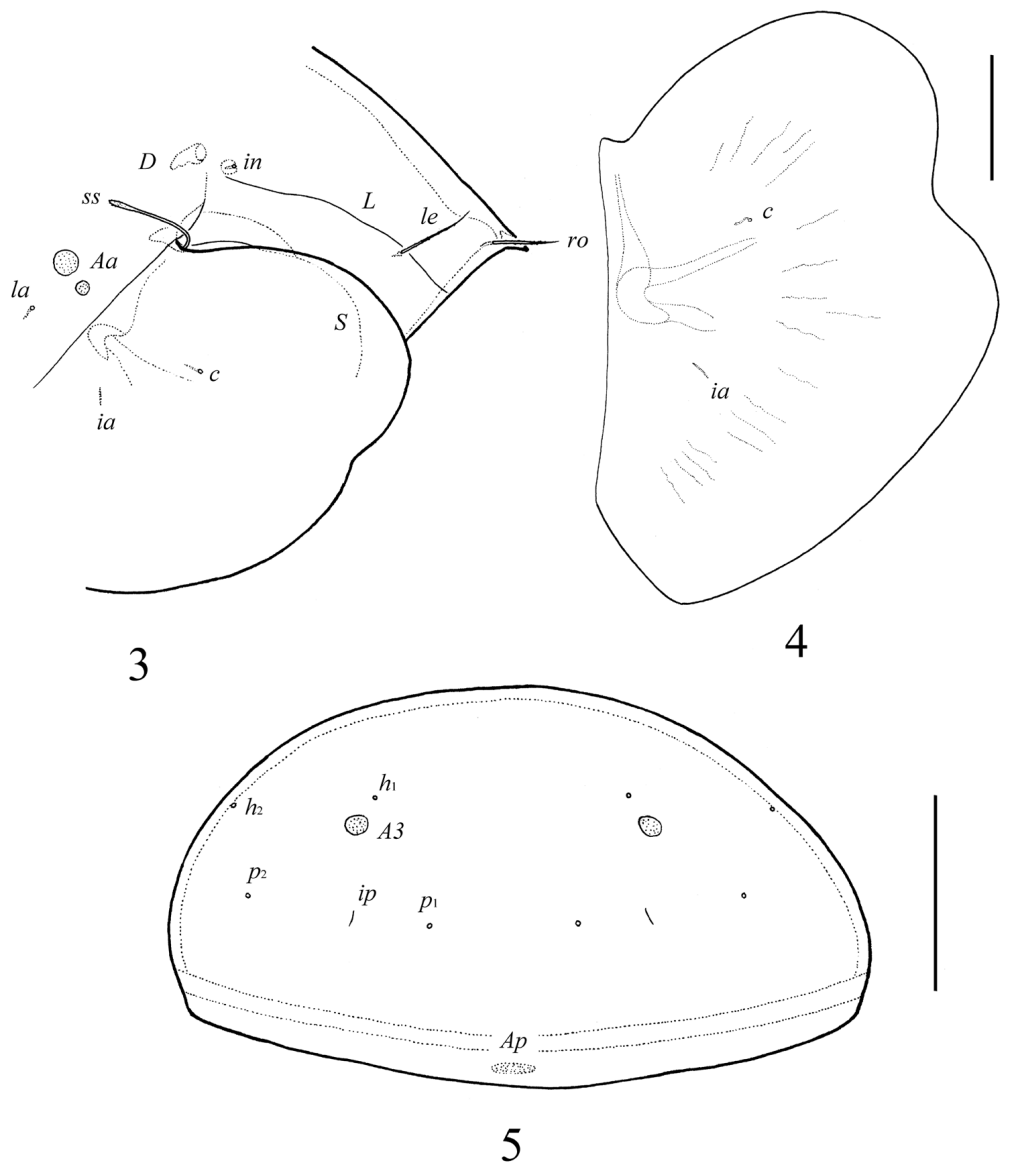
*Galumna tetraporosa* sp. n., adult: **3** dorso-lateral view of prodorsum and anterior part of notogaster and pteromorph **4** pteromorph **5** posterior view of notogaster. Scale bars 200 μm (**3, 5**), 100 μm (**4**).

**Table 1.** Leg setation and solenidia of adult *Galumna tetraporosa* sp. n. (same data for *Galumna granalata* Aoki, 1984).

Leg	Trochanter	Femur	Genu	Tibia	Tarsus
I	*v*’	*d*, *(l)*, *bv*’’	*(l)*, *v*’, σ	*(l)*, *(v)*, φ_1_, φ_2_	*(ft)*, *(tc)*, *(it)*, *(p)*, *(u)*, *(a)*, *s*, *(pv)*, *v*’, *(pl)*, *l*’’, ε, ω_1_, ω_2_
II	*v*’	*d*, *(l)*, *bv*’’	*(l)*, *v*’, σ	*(l)*, *(v)*, φ	*(ft)*, *(tc)*, *(it)*, *(p)*, *(u)*, *(a)*, *s*, *(pv)*, ω_1_, ω_2_
III	*v*’	*d*, *ev*’	*l*’, σ	*l*’, *(v)*, φ	*(ft)*, *(tc)*, *(it)*, *(p)*, *(u)*, *(a)*, *s*, *(pv)*
IV	*v*’	*d*, *ev*’	*d*, *l*’	*l*’, *(v)*, φ	*ft*’’, *(tc)*, *(p)*, *(u)*, *(a)*, *s*, *(pv)*

Roman letters refer to normal setae (ε to famulus), Greek letters to solenidia. Single prime (’) marks setae on anterior and double prime (’’) setae on posterior side of the given leg segment. Parentheses refer to a pair of setae.

#### Type deposition.

The holotype and one paratype are deposited in the collection of the Senckenberg Institution Frankfurt, Germany; one paratype is deposited in the collection of the Tyumen State University Museum of Zoology, Tyumen, Russia.

#### Etymology.

The specific name “*tetraporosa*” refers to the four notogastral porose areas *Aa*.

#### Remarks.

In having the pointed rostrum and bothridial setae with well developed head, *Galumna tetraporosa* sp. n. is most similar to *Galumna tokyoensis* Aoki, 1966 from the eastern Palaearctic region and *Galumna valida* Aoki, 1994 from the Pacific Islands. However, it clearly differs from both by the absence of interlamellar setae (versus long in *Galumna tokyoensis* and *Galumna valida*) and the presence of two pairs of notogastral porose areas *Aa* (versus one pair in *Galumna tokyoensis* and *Galumna valida*).

### Galumna granalata

Taxon classificationAnimaliaOribatidaGalumnidae

Aoki, 1984

Figs 6 – 10

#### Description.

*Measurements*. Body length: 431–464 (eight specimens); notogaster width: 332–356 (eight specimens).

*Integument*. Body color light brown. Body surface smooth. Pteromorphs with radiate wrinkles and small (length up to 12), elongate, grain-shaped tubercles. Wrinkles and tubercles very poorly visible in some specimens.

*Prodorsum*. Rostrum rounded. Rostral (28–32) and lamellar (16–20) setae setiform, thin, smooth, often not visible in dorsal view. Interlamellar setae minute (4). Bothridial setae (57–65) with long stalk and shorter, barbed, clavate head. Exobothridial setae absent. Porose areas *Ad* present, elongate oval (10–16 × 4–6). Lamellar and sublamellar lines parallel, curving backwards.

*Notogaster*. Anterior notogastral margin well developed, convex. Dorsophragmata of medium size, triangular. Notogastral setae represented by 10 pairs of alveoli. Four pairs of rounded porose areas with distinct borders: *Aa* (16–20) larger than *A1* (12–16), *A2* (8–10) and *A3* (10–16). Alveoli *la* inserted posteriorly to *Aa*. Lyrifissures *im* located between *lm* and *lp*. Opisthonotal gland openings located laterally to *A1*. Median pore present, located little posterior to the virtual line connecting porose areas *A1*.

*Gnathosoma*. Generally, morphology of subcapitulum, palps and chelicerae typical for most Galumnidae (for example, see Ermilov and Anichkin 2010, 2011; Ermilov et al. 2011). Subcapitulum little longer than wide (106 × 98). Subcapitular setae setiform, smooth; *a* (24) longer and little thicker than *m* (12) and *h* (16). Two pairs of adoral setae (10) setiform, slightly barbed. Palps (86) with setation 0–2–1–3–9(+ω). Solenidion straight, thickened, blunt-ended, attached to eupathidium. Chelicerae (131) with two barbed setae; *cha* (49) longer than *chb* (28). Trägårdh’s organ distinct.

*Epimeral and lateral podosomal regions*. Apodemes (1, 2, sejugal, 3) well visible. Three pairs of epimeral setae (*1a*, *3b*, *4a*) observed ventrally, all setiform, thin, smooth; *1a* and *3b* (12–16) longer than *4a* (8). Discidia triangular, circumpedal carinae distinct.

*Anogenital region*. Six pairs of genital setae (12–16) setiform, thin, smooth. Anterior edge of genital figs with three setae. One pair of aggenital (8), two pairs of anal (4) and three pairs of adanal (4) setae minute. Adanal setae *ad*_3_ inserted laterally to adanal lyrifissures *iad*. Postanal porose area (*Ap*) very small, rounded (6–12). Ovipositor of typical morphology for Galumnidae (Ermilov 2010): elongate, narrow (183 × 41). Length of lobes 77, length of cylindrical distal part 106. Setae setiform, smooth, ψ_1_ ≈ τ_1_ (36–41) longer than ψ_2_ ≈ τ_a_ ≈ τ_b_ ≈ τ_c_ (16–20). Six setae *k* short (6).

*Legs*. Generally, morphology of leg segments, setae and solenidia typical for most Galumnidae (for example, see Ermilov and Anichkin 2010, 2011; Ermilov et al. 2011). Formulae of leg setation and solenidia: I (1–4–3–4–20) [1–2–2], II (1–4–3–4–15) [1–1–2], III (1–2–1–3–15) [1–1–0], IV (1–2–2–3–12) [0–1–0]; homology of setae and solenidia indicated in Table 1.

**Figure 6.**
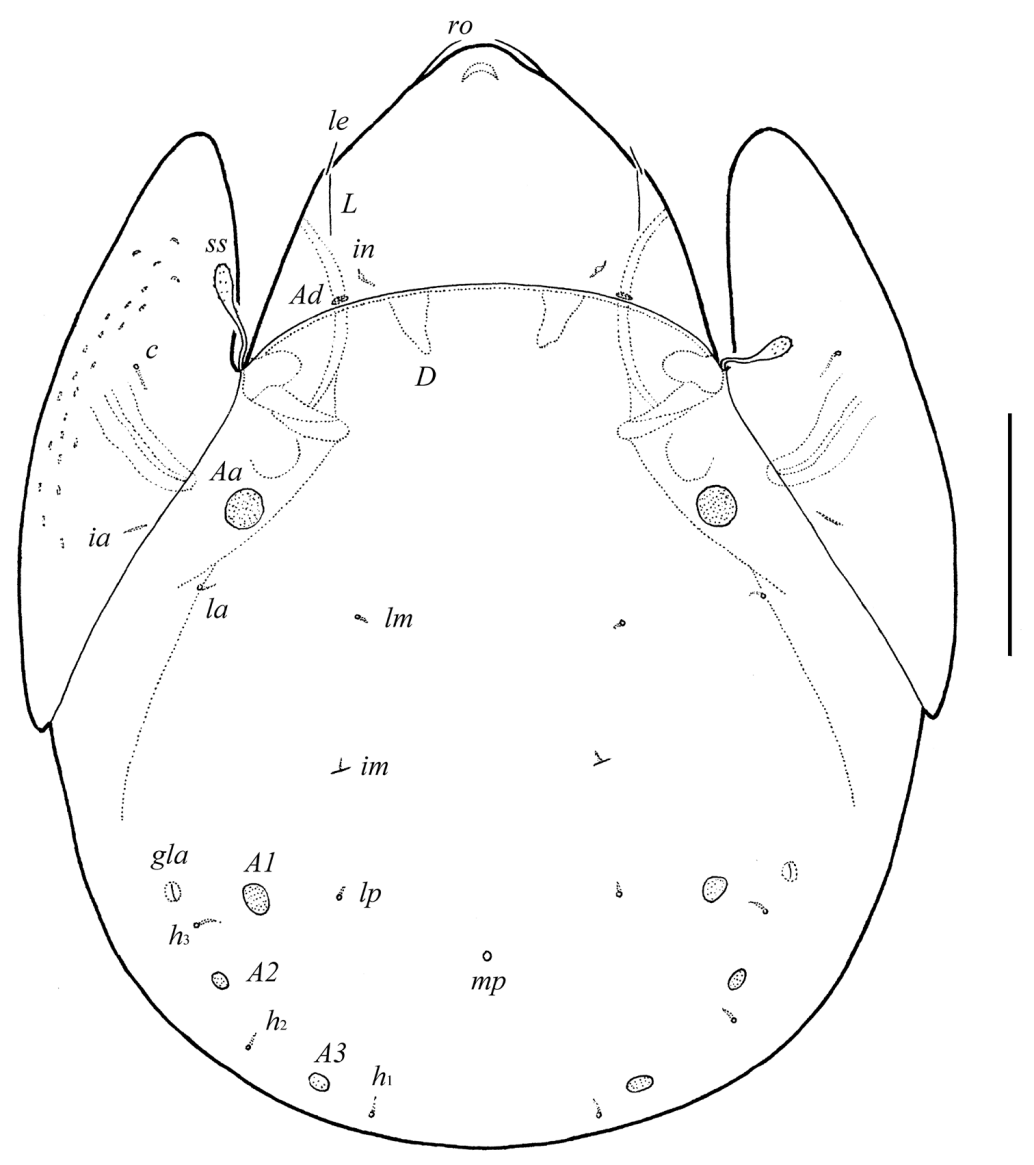
*Galumna granalata* Aoki, 1984, adult: dorsal view. Scale bar 100 μm.

**Figure 7.**
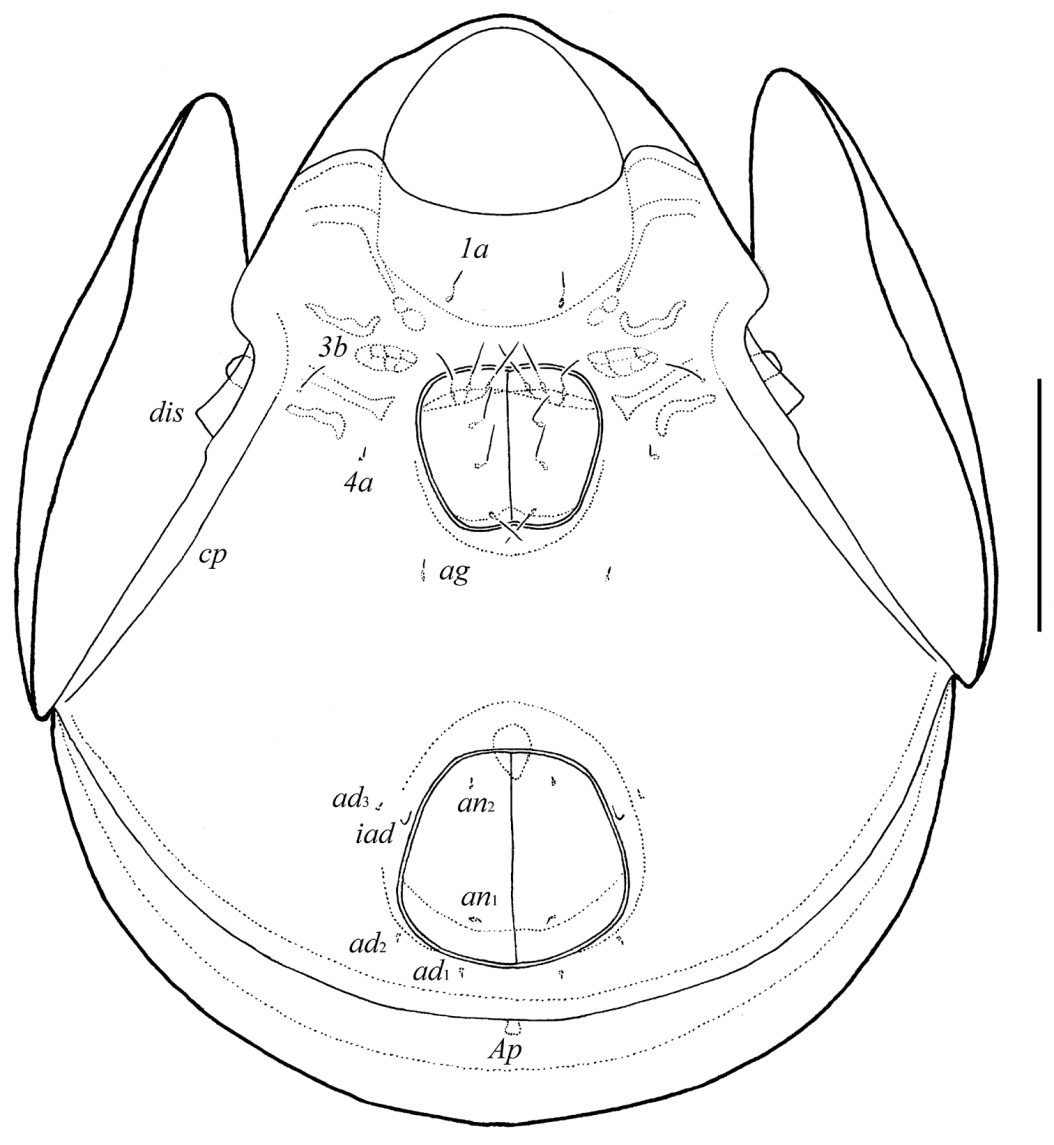
*Galumna granalata* Aoki, 1984, adult: ventral view (gnathosoma and legs not illustrated). Scale bar 100 μm.

**Figures 8–10.**
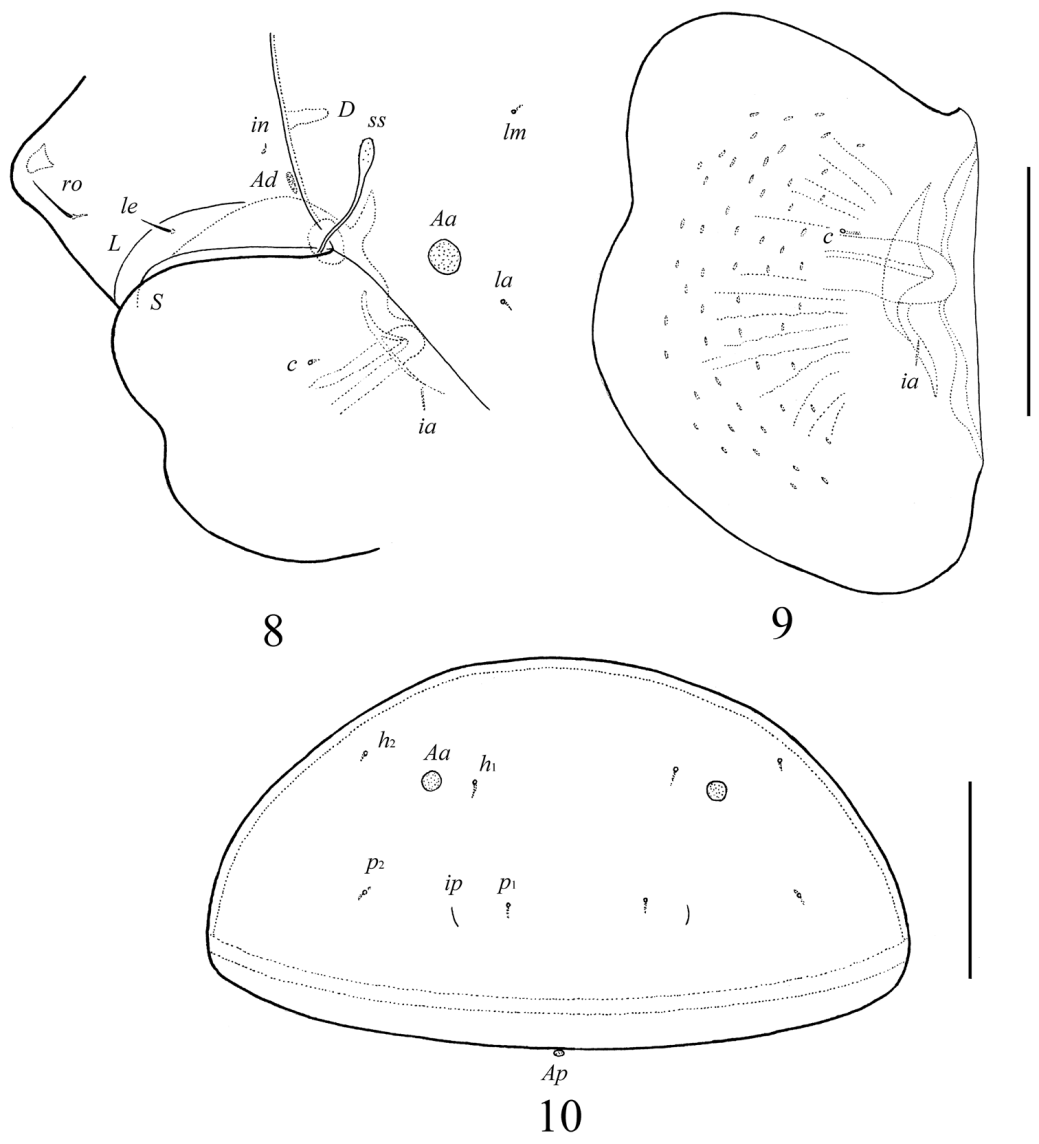
*Galumna granalata* Aoki, 1984, adult: **8** dorso-lateral view of prodorsum and anterior part of notogaster and pteromorph **9** pteromorph **10** posterior view of notogaster. Scale bar 100 μm.

#### Remarks.

*Galumna granalata* distinctly differs from other species of the genus *Galumna* by the presence of grain-shaped tubercles (granules in opinion of Aoki) on pteromorphs. The present Nepalese specimens of this species are morphologically and in general appearance similar to the Japanese specimens (Aoki 1984). Only one difference is that the body body size larger (431–464 × 332–356 versus 310–330 × 250–260 in Japanese specimens). We believe these differences represent intraspecific (e.g. geographical) variability.

### Notes on systematic placement of some *Galumna*

The analysis of literature on the *Galumna*-species has revealed an incorrect systematic placement of three species: *Galumna floridae* (Jacot, 1929), *Galumna hexagona* Balogh, 1960 and *Galumna mauritii* Mahunka, 1978.

*Galumna floridae* and *Galumna hexagona* were described by Jacot (1929, see also 1935; from U.S.A.) and Balogh (1960; from Angola), respectively. However, both species have a specific morphology of the notogaster (almost hexagonal, truncated posteriorly unlike other species of *Galumna* with well rounded notogaster posteriorly). Hexagonal and truncated posteriorly notogaster is a generic character of the genus *Notogalumna*. It was proposed by Sellnick (1959) with *Notogalumna praetiosa* Sellnick, 1959 as type species, and includes five species, which are distributed in the Oriental region, Tanzania, Polynesia and Seychelles (Subías 2004, online version 2014). Representatives of *Notogalumna* differ from *Galumna floridae* and *Galumna hexagona* also by the setiform bothridial setae (clavate in *Galumna floridae* and *Galumna hexagona*), fused porose areas *A1* and *A2* (versus not fused in *Galumna floridae* and *Galumna hexagona*) and localization of porose areas *Aa* dorsally (versus lateral localization close to hinge in *Galumna floridae* and *Galumna hexagona*). However, all listed characters are known in the other species in Galumnidae. For example, the large genera *Galumna* and *Pergalumna* Grandjean, 1936 include species with numerous variations of morphology of bothridial setae and localization of notogastral porose areas. Hence, in our opinion, inclusion of *Galumna floridae* and *Galumna hexagona* in the genus *Galumna* is incorrect. We suggest these species should be transferred to *Notogalumna*: *Notogalumna floridae* (Jacot, 1929) comb. n. and *Notogalumna hexagona* (Balogh, 1960), comb. n.

*Galumna mauritii* was described by Mahunka (1978) from Mauritius. However, this species has very short sublamellar lines; they are almost absent – see Fig. 59 in the original description (versus all species of *Galumna* with long, well developed sublamellar lines). Absence of sublamellar lines and localization of lamellar setae laterally to lamellar setae are the generic characters of the genus *Dimidiogalumna*. This genus was proposed by Engelbrecht (1972) with *Dimidiogalumna villiersensis* Engelbrecht, 1972 as type species, and includes four species, which are distributed in the Ethiopian region, Comoro Islands, Vietnam, central China and Japan (Subías 2004, online version 2014; Ermilov and Anichkin 2014). Hence, in our opinion, inclusion of *Galumna mauritii* in the genus *Galumna* is incorrect. We suggest this species should be transferred to *Dimidiogalumna*: *Dimidiogalumna mauritii* (Mahunka, 1978), comb. n.

## Supplementary Material

XML Treatment for Galumna tetraporosa

XML Treatment for Galumna granalata
